# The ligamentum mucosum’s potential as a preventative structure in the development of knee osteoarthritis

**DOI:** 10.1186/s40634-023-00681-1

**Published:** 2023-11-03

**Authors:** Alexandra Martin, Kenneth Liu, Majid Alimohammadi

**Affiliations:** 1 UBC (Vancouver, Canada) Biology, Vancouver, Canada; 2 UBC (Vancouver, Canada) Faculty of Medicine – Cellular and Physiological Sciences, Vancouver, Canada

**Keywords:** Ligamentum mucosum, Infrapatellar plica, Knee osteoarthritis, Synovial plica

## Abstract

**Purpose:**

This paper aimed to identify whether the presence, type, and/or morphology of the ligamentum mucosum could play a role in the development of knee osteoarthritis. Since its microscopic structure is alike that of other knee ligaments, it was hypothesized that its presence could facilitate knee motion and stability, thus preventing or reducing the extent of knee osteoarthritis.

**Methods:**

Thirty three cadavers (a total of 51 knees) were dissected. The ligamentum mucosum, if present, was measured with a digital caliber and a measuring tape in terms of length, width, and thickness. Knee osteoarthritis was assessed in six regions of the knee. The OuterBridge Classification System (Grades 0–4) was used to visually assess the extent, in addition to probing the area. Osteoarthritis was deemed present if the grade was 2 or greater.

**Results:**

The presence of the ligament was associated with a lower mean osteoarthritis level in the trochlear groove and lateral tibial plateau regions (*p* < 0.001 and *p* = 0.013, respectively). Overall osteoarthritis of the knee was also present at varying levels for each type of the ligamentum mucosum (*p* < 0.001). The patella and the medial condyle had the greatest levels of osteoarthritis, while the medial and lateral tibial plateaus had the lowest levels.

**Conclusion:**

The presence of the ligamentum mucosum is linked with decreased osteoarthritis in the trochlear groove region. In addition, both the absent ligament and its classification as a vertical septum are associated with increased knee osteoarthritis.

**Level of evidence:**

Five.

## Introduction

Knee osteoarthritis (KOA) is a highly prevalent, disabling, and multi-factorial joint disease. In the United States, a report by Lawrence et al. highlights that a fifth of Americans are living with the disease [1]. However, this value is likely too conservative: typically, KOA diagnoses succeed a patient presenting symptoms, but not all patients with KOA present any. Additionally, there are large inconsistencies in the diagnostic methods for KOA, which could lead to missed diagnoses [2]. Age, body max index (BMI), inflammation, and anterior cruciate ligament (ACL) reconstructions are all factors associated with a higher likelihood of developing KOA [3–6].

The ligamentum mucosum (LM) has been morphologically described in the literature, but none have investigated its potential function in knee osteoarthritis [7–9]. It originates from the intercondylar notch (ICN) and inserts into the infrapatellar fat pad (IPFP). Kim, Min & Kim consider five types: vertical septum, fenestra type, split type, separate type, and absent [9]. The vertical septum and fenestra type have been found to be associated with anterior knee pain (AKP) and can also complicate radiographic readings of the ACL [10, 11]. In clinical cases with AKP, its resection resulted in a significant reduction in perceived pain [10, 12, 13]. It may also be resected in ACL reconstruction surgeries to spare the IPFP, a structure frequently researched for its beneficial and detrimental role in AKP and KOA [14–23]. Studies describe the ligament’s microscopic anatomy to be like that of other ligaments in the knee, though with a smaller tensile strength, suggesting only a minor role in knee stabilization and/or knee mobility [24, 25].

Given what has been known of this ligament so far, it was hypothesized that its presence could reduce the rate of, or even prevent the development of knee osteoarthritis. This cadaveric study aims to identify the potential role the ligamentum mucosum may have on the development of osteoarthritis of the knee. The findings could ultimately benefit the diagnostic process of knee osteoarthritis and may cause to reconsider procedures of various knee surgeries.

## Methods

### Samples

The embalmed cadavers were available through the UBC Body Donation Program. There were 33 cadavers that fit the criteria for data collection: an eligible knee must have shown no evidence of knee replacement and must not have been previously dissected. 51 knees were dissected and used for data collection, 23 left and 28 right (18 bilateral dissections).

### Dissections

**Fig. 1:**
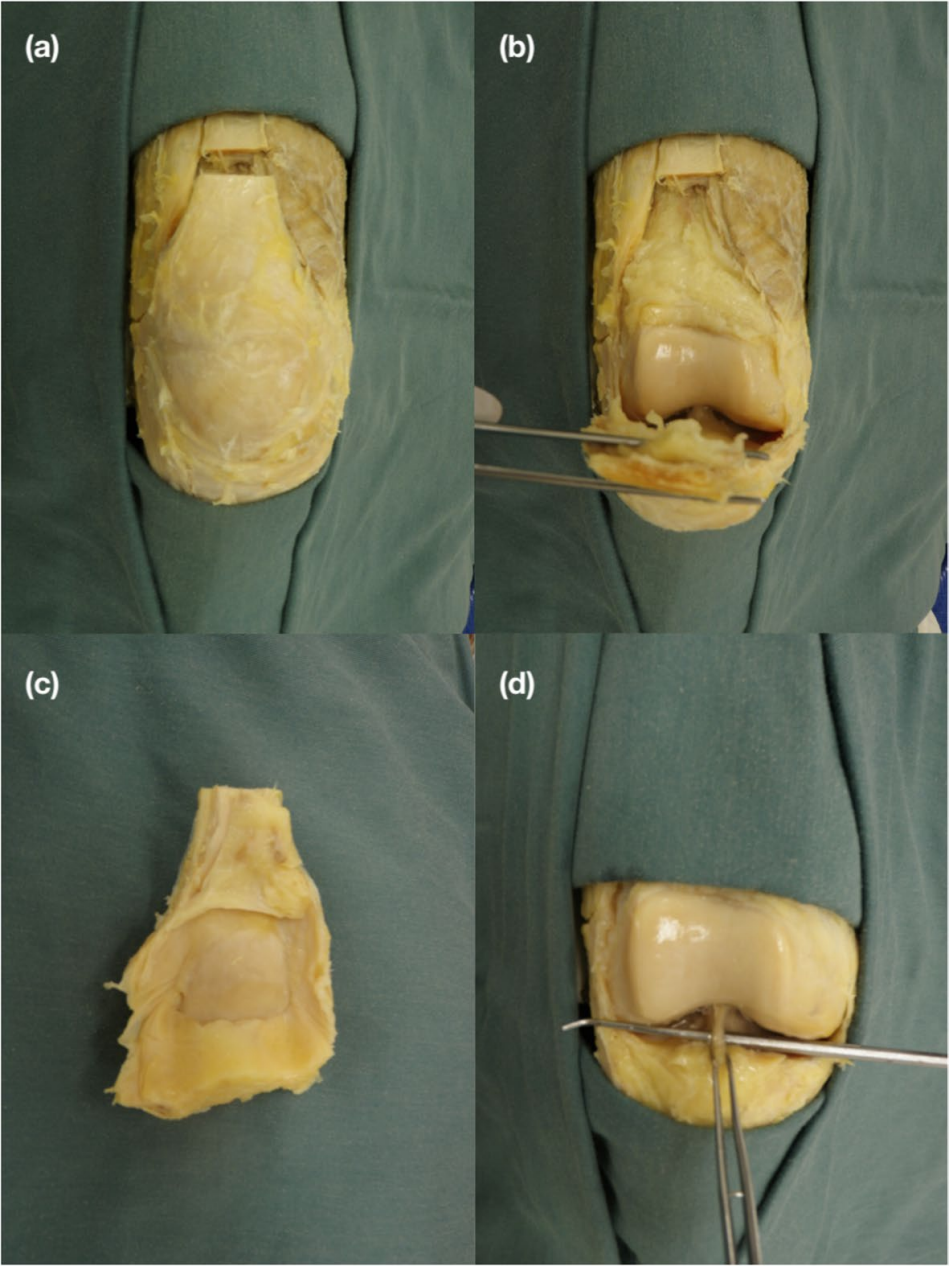
These steps follow the removal of the skin and subcutaneous tissue. **a** In a superior approach, the quadriceps tendon is cut about 5 cm above the patella. The incision is followed along the sides of the tendon until the menisci. **b** The patella is resected, and subsequently detached by a horizontal incision through the patellar tendon at the level of the infrapatellar fat pad (IPFP). **c** The posterior/articulating portion of the patella is preserved to assess osteoarthritis. **d** The ligament here is identified as separate, and the attachments (intercondylar notch (ICN), and the IPFP) can clearly be seen

In the supine cadaver, two horizontal and superficial incisions were made through the skin approximately 10 cm above and below the patella. The skin, fat and excess connective tissue was delicately removed, the quadriceps tendon was isolated and horizontally cut about 5 cm above the knee to detach it superiorly. A head-block was placed underneath the knee to elevate and angle it. The quadriceps tendon was pulled away from the thigh and knee, and any visible synovial capsule was severed. At the level of the IPFP, the patellar tendon was cut horizontally to isolate the patella and expose the interior of the knee capsule (Fig. 1).

### Data collection

If the ligament was present, its length was measured with a measuring tape, while its width and thickness (both at 3 points: proximal, middle, distal) were measured with a digital caliber (brand: FineSource, model: VC-HeiKa). The proximal point was as close to the ICN as possible, while the distal point was at the distal end of the ligament, where its borders start to lose definition as it inserts into the IPFP.

The extent of osteoarthritis was established using the OuterBridge Classification (OBC) System [26] in six regions of the knee: patella, trochlear groove, medial condyle, lateral condyle, medial tibial plateau, and lateral tibial plateau. The extent was inspected visually along with probing of the area.

### Statistical analyses

All statistical tests were performed on Microsoft Excel version 16.73. In the cases of bilateral observations, the quantitative morphological measurements of both knees were averaged to avoid pseudo-replicates for statistical tests that included demographic properties (i.e., sex, age, BMI). Analyses were considered significant when the p-value (including two-tailed p-value) was smaller than the critical *p* value = 0.05.

The Kruskal Wallis test was used to identify a difference in means between numerical variables across categories, that did not conform ANOVA’s requirement of similar sample and variance size. It was used to evaluate the relationship between osteoarthritis grade in each region and length of ligament; between the type of ligament and osteoarthritis grades in each region; and between the average width and thickness of the ligament and osteoarthritis grades in each. The ANOVA single factor test was used to identify a difference in means between numerical variables across categories that have equal sample sizes and variance. It was used to evaluate the average level of osteoarthritis between the various regions; between the width of different ligament types; and between the thickness of different ligament types. The two-sample t-test with unequal variance (two-tailed) was used to identify differences in the means of two categorical variables. It was used to evaluate the overall knee osteoarthritis level between the age between groups with or without the ligament; the BMI between groups with or without the ligament; and the levels of osteoarthritis in different regions between groups with or without the ligament. The Chi-contingency test was used to identify an association between two categorical variables. It was used to evaluate the relationship between the type of the ligament and the presence of osteoarthritis, and between the type of ligament and sex. The correlation test was used to identify a correlation between two numerical variables. Namely, between the presence of osteoarthritis (converted to binary) and the length of the ligament, and between the BMI and the length of the ligament. The Fisher’s exact test was used to identify an association between two categorical variables in cases when the frequencies were too small. Namely, between sex and the presence of osteoarthritis; and between sex and the presence of the ligament.

All tests that yielded significant results also underwent an effect size analysis, reported either as Cohen’s d (for t-tests) and f^2^ for ANOVA tests.

## Results

### Demographic data

There were 21 male cadavers and 12 female cadavers, and their average age, height, weight, and BMI are reported in Table 1. These demographic factors were not significantly associated with the presence of the ligament (*p* > 0.05), except for age: the individuals with the ligament were on average older than those without (*p* = 0.034), with a large effect size value of 0.87 (Cohen’s d). Sex was not associated with KOA, the presence of the ligament, nor the type of ligament present (*p* > 0.05).

**Table 1:** Demographic information (sex, age, height, weight, BMI) for the cadavers with a ligament (*n* = 22) and those without (*n* = 11). Note that for the weight, height and BMI, there was *n* = 22 with the ligament and *n* = 10 without the ligament

	Presence of ligament (*n* = 22)	Absence of ligament (*n* = 11)
Number of females (*n* = 12)	9	3
Number of males (*n* = 21)	13	8
Mean age (± SD) in years	85.50 (± 9.79)	77.18 (± 9.38)
Mean height (± SD) in inches	66.18 (± 3.52)	67.55 (± 2.61)
Mean weight (± SD) in pounds	147.41 (± 13.36)	152.80 (± 15.72)
Mean BMI (± SD)	23.74 (± 2.37)	23.58 (± 2.27)

Abbreviations: *BMI* Body mass index. *SD* Standard deviation

### Osteoarthritis and the ligament

**Fig. 2:**
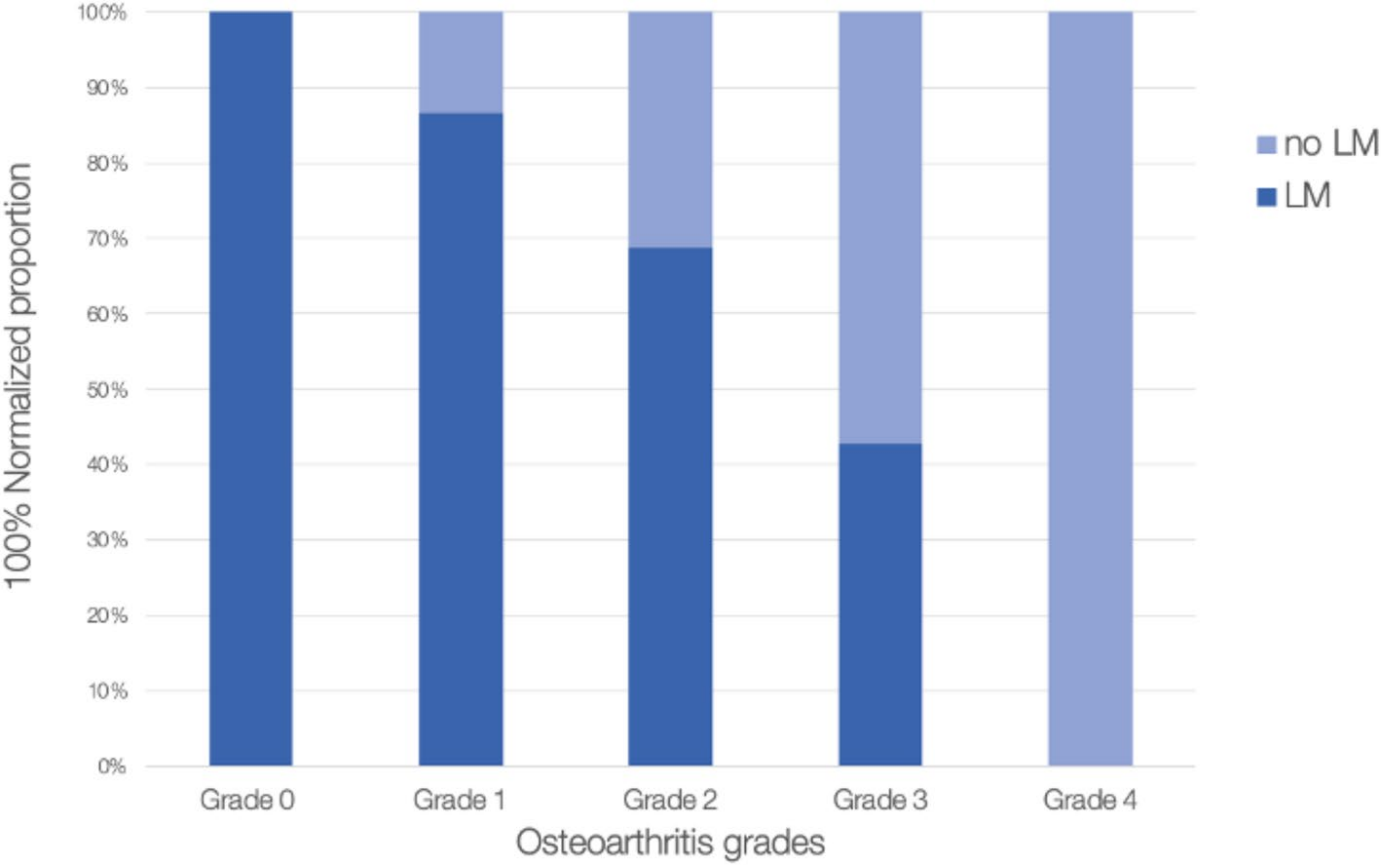
Normalized proportion of the knees with the ligament and those without for each grade of knee osteoarthritis in the trochlear groove (TG) region. There was a significant difference in the average osteoarthritis for those with and those without the ligament (*p* < 0.001)

**Fig. 3:**
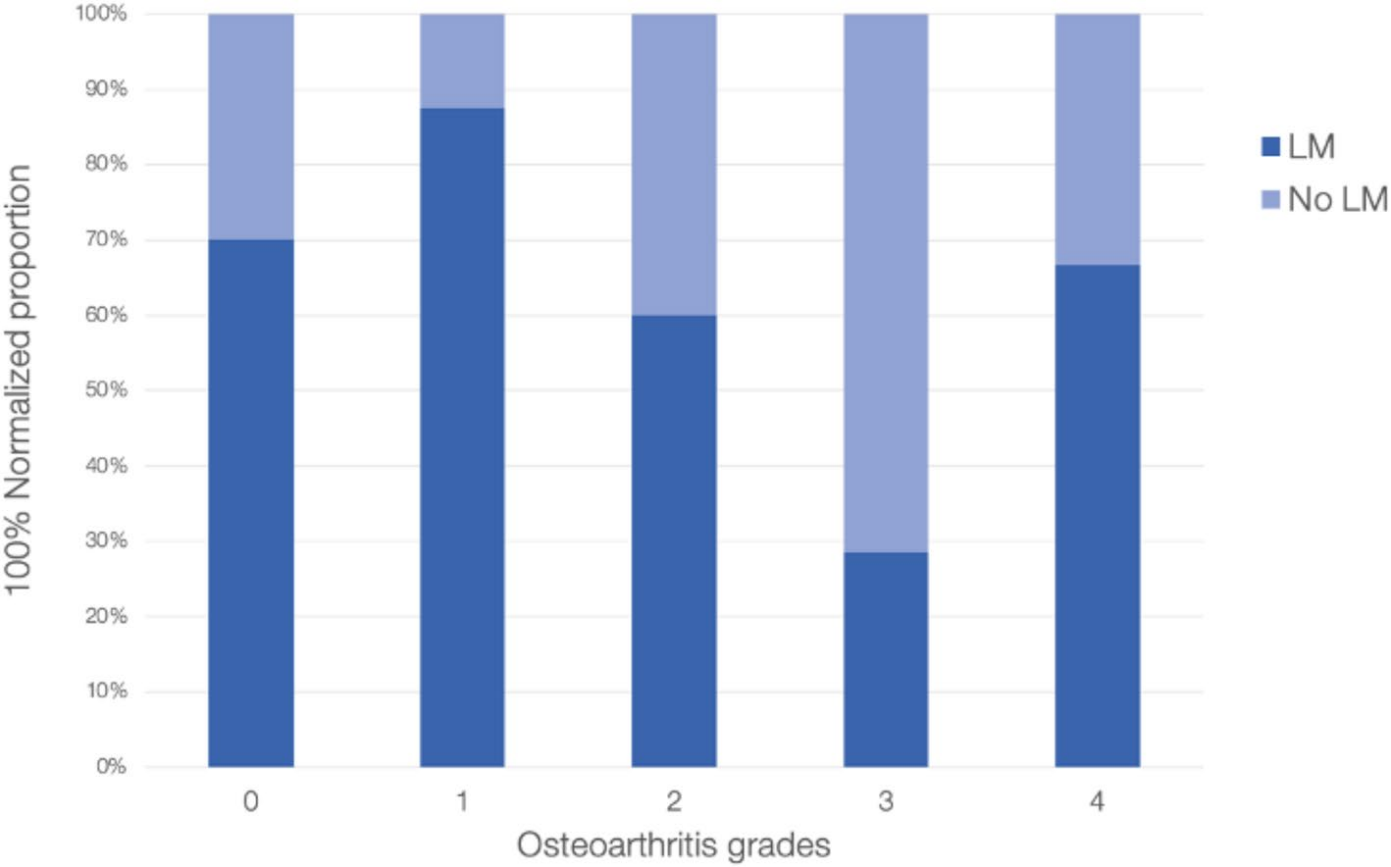
Normalized proportion of the knees with the ligament and those without for each grade of knee osteoarthritis in the lateral tibial plateau (LTP) region. There was a significant difference in the average osteoarthritis for those with and those without the ligament (*p* = 0.013)

There were significant differences in the mean osteoarthritis level between each regions (*p* < 0.001, f^2^ = 0.54). There were no significant differences in the average osteoarthritis level in most of the knee regions when the ligament was present except for the trochlear groove (*p* < 0.001) and lateral tibial plateau (*p* = 0.013) regions (Figs. 2 and 3). In the trochlear groove region, the average level of osteoarthritis was greater in the knees lacking the ligament, at 1.94 (median = 2), while in the knees with the ligament had an average osteoarthritic value of 1.35 (median = 1) (Cohen’s d = 1.13). In the lateral tibial plateau, there was some association between the average level of osteoarthritis and the presence (or absence) of the ligament. The knees lacking the ligament had an average osteoarthritic value of 2.59 (median = 3), while the knees with the ligament had an average value of 1.56 (median 1.5) (Cohen’s d = 0.92).

### Types and measurements of the ligament:

**Fig. 4:**
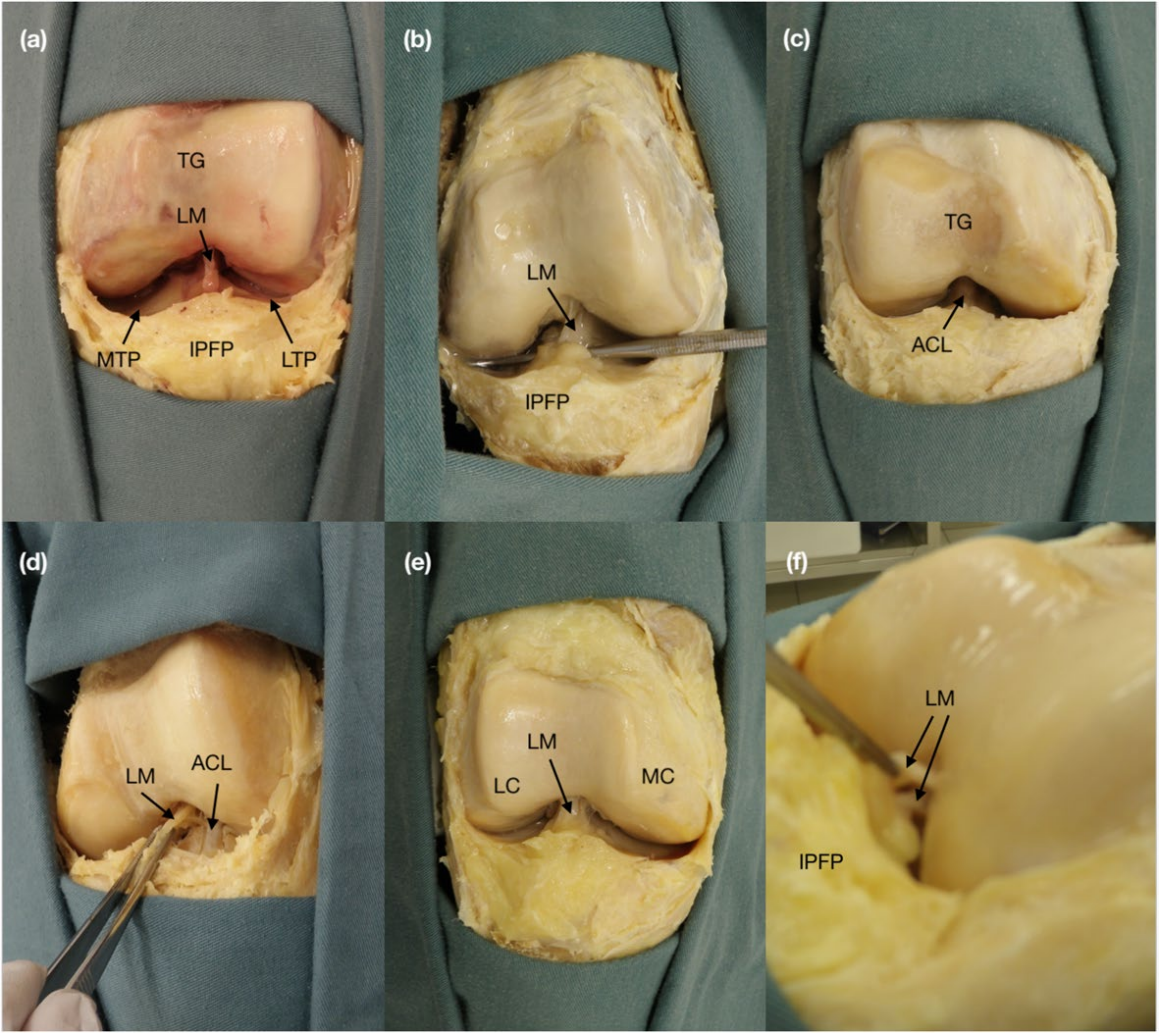
Types of the ligamentum mucosum identified. **a** Separate type. The ligament is a single and running from the ICN to the IPFP. **b** Vertical septum type. The ligament’s posterior surface is in direct contact with the ACL throughout its length. **c** Absent type. There is no ligament to obstruct the view of the ACL. **d** Fenestra type. The ligament is attached to the ACL posteriorly, with fenestrae between the two. **E–f** Split type, anterior and medial view. The ligament is made up of two distinct, separate and parallel bands, one sitting between its anterior counterpart and the ACL. Abbreviations: *ICN* Intercondylar notch. *IPFP* Infrapatellar fat pad. *TG* Trochlear groove. *LM* Ligamentum mucosum. *MTP* Medial tibial plateau. *LTP* Lateral tibial plateau. *ACL* Anterior cruciate ligament. *MC* Medial condyle. *LC* Lateral condyle

Five types were observed: separate (*n* = 17, 33.3%), vertical septum (*n* = 11, 21.6%), split (*n* = 3, 5.9%), fenestra (*n* = 3, 5.9%) and absent (*n* = 17, 33.3%) (Fig. 4). Table 2 highlights the average width, length, and thickness of the ligament for each of its type (except absent). The types of the ligament were not associated with varying levels of osteoarthritis, nor with the presence of osteoarthritis in any specific region. However, as a whole, some types were associated with the presence of knee osteoarthritis (χ^2^(5, *n* = 33) = 10.9802, *p* = 0.03, Cohen’s d = 0.57): knees with the fenestra or absent type all presented some degree of knee osteoarthritis (100%), while the split and vertical septum saw rates of 50% and 23% without any knee osteoarthritis, respectively.

**Table 2:** Average length, width and thickness of the ligament given its type

	Fenestra type (*n* = 3)	Separate type (*n* = 17)	Vertical septum (*n* = 11)	Split (*n* = 3)	Average (*n* = 34)
Width (± SD) in mm					
Proximal	4.70 (± 1.14)	3.86 (± 2.89)	3.56 (± 1.52)	4.03 (± 0.64)	3.85 (± 2.19)
Middle	4.80 (± 1.13)	3.11 (± 1.39)	3.53 (± 1.52)	3.67 (± 1.46)	3.44 (± 1.42)
Distal	4.37 (± 1.46)	3.25 (± 1.40)	3.60 (± 1.15)	3.93 (± 1.50)	3.52 (± 1.30)
Length (± SD) in mm	15.33 (± 0.58)	15.71 (± 3.74)	15.73 (± 3.10)	13.67 (± 3.51)	15.5 (± 3.24)
Thickness (± SD) in mm					
Proximal	1.03 (± 0.93)	0.48 (± 0.29)	1.05 (± 0.90)	1.17 (± 0.32)	0.77 (± 0.65)
Middle	1.03 (± 0.93)	0.49 ± 0.25)	1.09 (± 0.96)	1.13 (± 0.32)	0.79 (± 0.67)
Distal	1.20 (± 0.98)	0.55 (± 0.28)	1.14 (± 0.99)	1.20 (± 0.56)	0.86 (± 0.70)

Abbreviation: *SD* Standard deviation

There was a very weak negative correlation between the length of the ligament and the overall presence of osteoarthritis (*r* =—0.1668), though there were no significant differences in mean lengths of the ligament and the level of osteoarthritis in each of the knee regions. There were also no significant differences in average width or thickness for various osteoarthritis levels in each of the six regions of the knee (*p* > 0.05). There was a negligible correlation between the length of the ligament and BMI (*r* =—0.0893), and there was no association between the length of the ligament and a particular type (*p* > 0.05).

## Discussion

The key findings of this current study include the associations between the lack of KOA and the split and vertical septum types of ligament, as well as between a reduced KOA and the presence of the ligament in the trochlear groove and lateral tibial plateau regions. Investigating all the possible roles (whether beneficial or detrimental) the ligamentum mucosum, coupled with the IPFP, has on knee degeneration, knee biomechanics, and AKP should be an important focus of research. These may impact diagnostic decisions and accuracy and could call to restructure and modify current knee surgical procedures.

At the present time, several models for the morphological assessment of the ligamentum mucosum have been suggested. This current study supports Kim, Min & Kim’s identification of the five different types of the ligamentum mucosum, though in different frequencies [9]. The separate type was present in 33.3% of the knees (vs. 60.5% in Kim, Min & Kim’s study); the vertical septum type was present in 21.6% of the knees (vs. 10.5%); the split type was present in 5.9% of the knees (vs. 13.5%); the fenestra type was present in 5.9% (vs. 1.0%); and the absent type was present in 33.3% of the knees (vs. 14.5%).

As Kim, Min & Kim described, the split type has two attachments in each of the ICN and the IPFP [9]. Its demarcating feature is a longitudinal fissure, resulting in two bands sitting anteriorly to the ACL, one band posterior to the other. The vertical septum type is a complete, un-punctured ligament that attaches to the ACL along its posterior length. These two types may be associated with a lower KOA grade compared to the separate and fenestra type due to physics. Since the separate type is a single band, and the fenestra type is like the vertical septum except with multiple perforations (‘fenestrations’) throughout its length, it may concern the strength of the ligament. Studies have described the ligamentum mucosum to be of the same microscopic anatomy as that of a true ligament though with weaker tensile strength, but no description as to the type of ligament studied was included in the report [13, 24]. It could be that the vertical septum and split types provide additional support through more balanced weight distribution along the length (vertical septum) and through dividing the load between the two bands (split).

The results indicate that the patellar-TG osteoarthritis is particularly advanced and seems to spread to either condyle as a result of dysplasia. In the TG, there was a significant difference in average osteoarthritis level between the knees with and without the ligament. Figure 2 suggests that osteoarthritis decreases due to the presence of the ligament: grade 0 (no osteoarthritis) was 100% made up of knees with the ligament, and grade 4 (most advanced) was 100% made up of knees without the ligament. The ligament may provide a cushion that supports the patella in locomotion, reducing the friction between the patella and TG.

The LTP saw the lowest average osteoarthritis level (along with the MTP) (Fig. 3). Further, the data suggest a significant difference in average osteoarthritis of the LTP between the knees with and without the ligament. Though negligible, the tensile strength of the ligament described by Norris et al. could vertically support the joint and reduce overall load particularly in the highly articulating lateral aspect of the plateau [24, 27]. However, Fig. 4 does not show the same relationship as for the TG. The average ratios of knees with and without the ligament for each osteoarthritis grade are more inconsistent and variable. This pattern may be due to the nature of the LTP itself. The LTP is meant to handle more overall load and downward force; as such, chondral lesions may not be the ultimate sign of osteoarthritis in this region. Instead, MRIs and/or radiographic images should be used to observe the JSN.

Kim & Choe had importantly identified fibrosis and inflammation as key processes leading to overall KOA [12]. We could expect thus expect arthritic knees to have a LM that is thicker than their non-arthritic counterparts. This study’s results suggested no significant difference in means between these. This discrepancy may be attributed to the difficulty of measuring the thickness for the fenestra and vertical septum type. Or, these data may suggest that the ligament itself does not reflect the level of KOA, unlike the IPFP that does [16, 18–21, 28].

A key limitation was the method of osteoarthritis assessment. Firstly, the investigator collecting the data was consistent, but not officially trained in the matter. Additionally, the scale used may not have been the best tool. The OBC System is best used for early OA cases but was used here for mild to advanced assessments. For future studies, adapting the OBC System to cadavers should be done by replacing softness to pinkness (and its intensity).

The large number of samples was an asset in this cadaveric study. However, when separated into distinct groups, the sample sizes were often too small and resulted in important effect size values. As stated in most of the literature, the ligamentum mucosum is a remnant from embryological development [8, 9, 24]. As such, we should expect the incidences of the ligament to decrease as the age increases, but this current study suggests the opposite. This discrepancy with the literature is most likely due to some sampling error: the group without the ligament (average age: 77 years) had a sample size of 22, while the group with the ligament (average age: 86 years) had a sample size of 11. Indeed, the effect size was calculated to be 0.86, a value that strongly suggests that the sample size may have impacted the results and statistical association.

## Conclusion

Elucidating the role of the ligamentum mucosum in knee osteoarthritis is of great clinical relevance as it could pave the way to enhanced diagnostic methods and treatments of the disease. This current study shows that when the ligamentum mucosum is absent, the knee presents a higher overall osteoarthritis level, particularly in the trochlear groove region. When present, the ligament’s type seems to be associated with the extent of knee osteoarthritis. Indeed, lower overall knee osteoarthritis grades seem to accompany the split and vertical septum types, whereas the absent ligament is most often seen alongside advanced overall osteoarthritis levels in the knee.

## Data Availability

The datasets generated and analysed in this current study are not publicly available due to the lack of representation of the sample. They may be available from the corresponding author on reasonable request.
